# Suboptimal concordance in testing and retesting results of triple-negative breast carcinoma cases among laboratories: one institution experience

**DOI:** 10.1186/s12935-019-0987-7

**Published:** 2019-10-11

**Authors:** Jose De Jesus, Marilin Rosa

**Affiliations:** Department of Anatomic Pathology, H. Lee Moffitt Cancer Center and Research Institute, 12902 Magnolia Drive, Tampa, FL 33612 USA

**Keywords:** Concordance testing, Dual in situ hybridization, Estrogen receptor, Fluorescence in situ hybridization, Human epidermal growth factor receptor 2 immunohistochemistry, In situ hybridization, Progesterone receptors, Triple-negative breast carcinoma

## Abstract

**Background:**

Triple-negative breast carcinoma (TNBC) patients do not benefit from hormone- or human epidermal growth factor receptor 2- (HER2-) targeted therapies. Accurate testing is pivotal for these patients.

**Methods:**

TNBC cases that were retested at our institution during a 3-year period were evaluated for concordance rates in estrogen (ER) and progesterone (PR) receptor and HER2 results.

**Results:**

We found 19 (22%) discrepancies (13 major/6 minor) among 86 cases. Minor discrepancies were in HER2 changes by immunohistochemistry, and all cases were demonstrated to be negative by and dual in situ hybridization. All major discrepancies were in ER/PR expression changes. In only 2 cases the treatment changed based on repeated results and/or patient history.

**Conclusions:**

Discrepancies in prognostic/predictive testing continue to be frequent despite rigorous regulations. However, since for the majority of patients in our setting, the treatment plan did not change, reflex retesting for TNBC has been deemed unnecessary in our institution.

## Background

Estrogen (ER) and progesterone (PR) receptors and human epidermal growth factor receptor 2 (HER2) are the classic tumor markers for breast carcinoma with a direct effect on treatment decisions [1, 2]. By definition, triple-negative breast carcinomas (TNBCs) lack ER and PR and HER2 expression. TNBCs are usually of high histologic grade, affect a younger population, and carry a poor prognosis [3–6]. In addition, TNBCs are heterogeneous and comprise several histologic subtypes and unique patterns of gene expression, further complicating diagnosis and treatment [4].

Patients with TNBC do not benefit from hormonal or HER2-targeted therapies [7]. Therefore, combined surgery, cytotoxic chemotherapy, and radiation therapy are often their main treatment options [4]. Neoadjuvant chemotherapy is frequently offered to patients with TNBC, as studies have consistently reported neoadjuvant therapy as having a higher response rate among patients with TNBC than patients non-triple negative breast carcinoma. Furthermore, pathologic complete response (pCR) has been shown to predict long-term outcomes and subsequent disease-free and overall survival among patients with TNBC [3, 7, 8]. Thus, accurate identification of TNBC is necessary to ensure adequate patient treatment management, better treatment planning, lower costs, and avoid patient exposure to unnecessary and potentially harmful treatments.

In current laboratory practice, testing for ER, PR, and HER2 expression has become one of the most rigorously controlled techniques. To guarantee accuracy and decrease variability among laboratories, the American Society of Clinical Oncology (ASCO) and the College of American Pathologists (CAP) have established guidelines for testing standardization, specimen handling, and reporting [9, 10]. Despite the establishment of these guidelines, variability in results and interpretations of tests is still observed. We designed this retrospective study to evaluate concordance in testing for ER, PR, and HER2 expression in TNBC cases between laboratories, to assess whether repeating these markers offers any clinical benefit.

## Methods

This study was approved by the H. Lee Moffitt Cancer Center and Research Institute (MCC) Institutional Review Board. It included all cases of patients who had come to MCC for second opinions or case reviews, whose tissue samples were retested for ER, PR, and HER2 expression during a 3-year period, from January 2014 to December 2016, using tissue blocks and unstained slides from outside laboratories. In all cases, testing of these markers was repeated for clinical purposes. At MCC, every patient with a primary diagnosis of breast carcinoma from an outside institution was required to undergo secondary pathologic review and diagnosis confirmation before treatment. At the request of our breast cancer clinical program, we retested all recent cases that had been classified as triple negative at other institutions for which ER, PR, and HER2 slides were unavailable for confirmation. Recent cases included ones from patients who had not received any therapy after diagnosis. At MCC, HER2 status can be assessed using both immunohistochemistry (IHC) and dual in situ hybridization (DISH). Our laboratory performs approximately 2400 prognostic-/predictive-factor tests per year.

We retrospectively studied outside and MCC marker results, laboratory types (if available), testing methodologies used, and results discrepancies. Although a focused update was published in May 2018, which centered on HER2 patterns less commonly seen in practice, this update was unavailable at the time this study was conducted [11]. Therefore, the previous 2013 HER2 guidelines were used [9, 10]. Discrepancies were classified as minor or major according to their possible impacts on patients’ clinical treatment management. Major discrepancies were in changes from negative to positive or vice versa. Minor discrepancies included HER2 equivocal cases (outside versus MCC results), because these cases required testing using a second methodology. Score changes from 0 to 1 + and vice versa were also regarded as minor, as each is considered to be negative.

In our laboratory, the ER and PR antigens were analyzed with the Ventana system, using anti-ER (SP1) and anti-PR (1E2) rabbit monoclonal primary antibodies in sections of formalin-fixed paraffin-embedded tissue. The reference ranges for IHC results followed the guidelines established by the ASCO/CAP panel. The designations of ER or PR positive required ≥ 1% of tumor cells to be immunoreactive. The designations of ER or PR negative required < 1% of tumor cells to stain for ER or PR [10]. HER2 receptor protein expression by IHC was analyzed with the Ventana PATHWAY system, using an anti-HER2/neu (4B5) rabbit monoclonal primary antibody and Ventana iVIEW DAB Detection Kit. Positive HER2 (score of 3 +) was defined as intense, complete, and circumferential staining in > 10% of invasive tumor cells. Equivocal HER2 expression (2 +) was defined as circumferential membrane staining that is either incomplete and/or weak/moderate in > 10% of invasive tumor cells or complete, intense, and circumferential membrane staining in ≤ 10% of invasive tumor cells. HER2-negative status was defined as either incomplete membrane staining that is faint/barely perceptible in > 10% of invasive tumor cells (1 +) or absent or incomplete (faint/barely perceptible) membrane staining in ≤ 10% of invasive tumor cells (0) [9].

HER2 gene amplification was tested using the Ventana INFORM HER2 Dual ISH DNA Probe Cocktail assay. Using a 40× and/or 60× objective, in situ hybridization (ISH) was performed to analyze areas of invasive carcinoma cells. For each nucleus, we used bright-field microscopy to manually count the number of HER2 signals and the number of centromere 17 (CEP17) signals. HER2 gene status was reported as a function of the ratio between the average number of HER2 gene copies and the average number of Chr17 copies. The reference ranges for interpretation followed the ASCO/CAP panel guidelines. Non-amplified (negative) HER2 DISH was defined as HER2/CEP7 ratio < 2.0, with an average number of HER2 copies < 4.0 signals/cells. Amplified (positive) HER2 was defined as a HER2/CEP17 ratio ≥ 2.0 or < 2 with an average HER2 copy number ≥ 6.0 signals/cells. Equivocal HER2 expression was defined as a HER2/CEP17 ratio < 2.0 with an average HER2 copy number ≥ 4.0 and < 6.0 signals/cells [9].

## Results

During the 3-year period, 540 review cases were retested for ER, PR, and HER2 expression at MCC. Of those, 92 cases had been classified as TNBC at outside laboratories. Six cases were excluded from our study because the marker analyses were repeated using different tissue samples. Eighty-six cases were included in the study, of which 67 (78%) were core needle biopsies, 18 (21%) were resections, and 1 (1%) was a fine-needle aspiration biopsy. Three cases classified as HER2 equivocal by outside laboratories were also included in the study. Most cases were invasive ductal carcinomas of no special type (Table 1). At outside laboratories, testing for HER2 was performed exclusively by IHC in 56 cases and was only performed by fluorescence in situ hybridization in 8 cases; both methodologies were used in 22 cases. At MCC, HER2 was tested by IHC in all 86 cases, and both IHC and DISH methodologies were used in 79 cases. Nineteen discrepancies (22% of cases) affecting 18 patients were identified. Thirteen discrepancies were major (15% of cases) and 6 were minor (7% of cases).

**Table 1:** Histologic diagnosis of all cases included in the study

Diagnosis	Cases, no. (%)^a^
Invasive ductal carcinoma	72 (84)
Metaplastic carcinoma	6 (7)
Invasive lobular carcinoma	3 (3)
Invasive mucinous carcinoma	3 (3)
Metastatic site	2 (2)
Total	86

^a^Whole number introducing rounding error

### Major discrepancies

There were 12 cases with 13 major discrepancies. All major discrepancies involved ER and PR hormone receptor results. Among these, 4 were in the ER results of 4 different cases, 7 were in the PR results of 7 different cases, and 1 was in both the ER and PR results of 1 case (Table 2). Eight discrepancies (62% of discrepant cases) were in cases that had been originally tested at large commercial/reference laboratories. From the other 5 cases (38% of discrepant cases), 3 were originally tested at community laboratories and 2 were international cases.

**Table 2:** Twelve cases with 13 major discrepancies

Case ID	Diagnosis	Outside lab antibody	Laboratory	ER NEG/POS (%)^a^		PR NEG/POS (%)^a^	
				Outside	In-house	Outside	In-house
1	IDC	Unknown	International	NEG (< 1)	NEG (0)	NEG (< 1)	POS (1)
2	IDC	ER-monoclonal mouse antibody, clone ID5-α PR-monoclonal mouse antibody, clone PgR 636	Reference	NEG (0)	POS (3)	NEG (0)	NEG (0)
3	ILC	ER-GF11, Leica PR-1294, DAKO	Community	NEG (0)	NEG (< 1)	NEG (0)	POS (11)
4	IDC	ER-SP1 monoclonal, Ventana PR-1E2 monoclonal, Ventana	Community	NEG (0)	NEG (0)	NEG (0)	POS (1)
5	IDC	ER-SP1 monoclonal, Ventana PR-1E2 monoclonal, Ventana	Community	NEG (0)	POS (1)	NEG (0)	NEG (0)
6	IDC	ER-SP1 monoclonal, Ventana PR-1E2 monoclonal, Ventana	Community	NEG (< 1)	NEG (0)	NEG (< 1)	POS (5)
7	IDC	Unknown	Reference	NEG (< 1)	POS (2)	NEG (0)	NEG (0)
8	IDC	ER-SP1 monoclonal PR-1E2 monoclonal	Community	NEG (0)	NEG (0)	NEG (0)	POS (2)
9^b^	IDC	ER-SP1 monoclonal PR-1E2 monoclonal	Reference	NEG (< 1)	POS (2)	NEG (< 1)	POS (5)
10	IDC	ER-Monoclonal 6F11 PR-monoclonal mouse antibody, clone PgR 636	Community	NEG (< 1)	POS (15)	NEG (< 1)	NEG (< 1)
11	IDC	ER-SP1 monoclonal PR-1E2 monoclonal	Community	NEG (0)	NEG (0)	NEG (0)	POS (5)
12	IDC	Unknown	International	NEG (< 1)	NEG (0)	NEG (< 1)	POS (10)

*ER* estrogen receptor, *IDC* invasive ductal carcinoma, *ILC* invasive lobular carcinoma, *NEG* negative, *POS* positive, *PR* progesterone receptor

^a^Percentage of cells that stained for ER or PR

^**b**^Discrepancy in both ER and PR

All 6 minor discrepancies were in the HER2 results of 6 cases. In 4 (67%) of these discrepant cases, the discrepancies were in IHC only; in 1 case in ISH (fluorescence in situ hybridization versus DISH) only; and in 1 other case in both IHC and ISH. In 3 of these cases, IHC was changed from 2 + to 1 + (equivocal to negative); in 1 case, 2 + to 0 (equivocal to negative); and in 1 other case, 1 + to 2 + (negative to equivocal). There were 2 cases in which ISH was changed from equivocal to negative (Table 3).

**Table 3:** Six cases with 6 minor discrepancies

Case ID	Diagnosis	Outside results		MCC Results	
		HER2 (IHC)	HER2 (FISH)	HER2 (IHC)	HER2 (DISH)
1	IDC	1 +	NP	2 +	Not amplified
2	Bone met	2 +	NP	0	Not amplified
3	IDC	2 +	Not amplified	1 +	Not amplified
4	IDC	2 +	Equivocal	1 +	Not amplified
5	IDC	2 +	NP	1 +	Not amplified
6	IDC	1 +	Equivocal	1 +	Not amplified

*Bone met* bone metastasis, *IDC* invasive ductal carcinoma, *IHC* immunohistochemistry, *DISH* dual in situ hybridization, *FISH* fluorescence in sit hybridization, *NP* not performed, *MCC* Moffitt Cancer Center

## Discussion

TNBCs comprise approximately 12% to 25% of all invasive breast carcinomas [3–6]. These tumors are histologically characterized by high nuclear grade, frequent mitosis, and zonal necrosis. At presentation, there are no specific features that separate TNBC from other types of breast cancer; however, TNBC more often affects younger patients, especially those in the African-American population [3, 4]. TNBCs are considered clinically aggressive and have a high rate of recurrence, especially within the first 3 years after diagnosis [3]. Because patients with TNBC do not respond to hormonal- or HER2-targeted therapies, cytotoxic neoadjuvant therapy is often offered to them [6]. It has been shown that patients with TNBC who achieve pCR have excellent survival rates. However, patients with residual disease after neoadjuvant chemotherapy have considerably shorter overall and post-recurrence survival than patients with hormone receptor-positive tumors and partial response to treatment [3]. Therefore, it is particularly important to accurately determine ER, PR, and HER2 tumor status.

In 2010, the ASCO and CAP International Expert Panel recommended considering endocrine therapy for patients with breast tumors that express at least 1% ER-/PR-positive cells [10]. This recommendation was a change from the previously accepted optimum cut points that were established in the 1970s. With this change, the low-positive category of ER tumors emerged.

Multiple factors influence the accuracy of testing for ER, PR, and HER2, including specimen type, ischemic time, fixation time, fixative type, antibody selection, and determination of thresholds for positive results [12–14]. Since the implementation of the ASCO/CAP guidelines, our understanding of these variables has expanded significantly, leading to rigorous pre-analytic, analytic, and post-analytic standardization [10]. Laboratory volumes and testing practice variabilities have also been cited as factors that influence testing accuracy [15, 16]. Variability in concordance between central and local laboratories has been reported in several clinical trials that required central retesting for confirmation [12, 17, 18]. Furthermore, several published studies have compared testing for ER, PR, and HER2 expression among local and central (high-volume) laboratories [19, 20]. In separate studies, Paik et al. and Roche et al. found HER2 testing discordance rates to be as high as 18% and 26%, respectively, between local and central laboratories [18, 21]. In a previous study, we found a 32% discrepancy rate in HER2 cases that were retested at our institution [22]. Similarly, other studies have reported discrepancies in hormone-receptor testing [23, 24]. Layfield et al. [13] reported discrepancies in 26% of ER testing results, and our own data show a 15% discrepancy rate among TNBC cases.

**Fig. 1:**
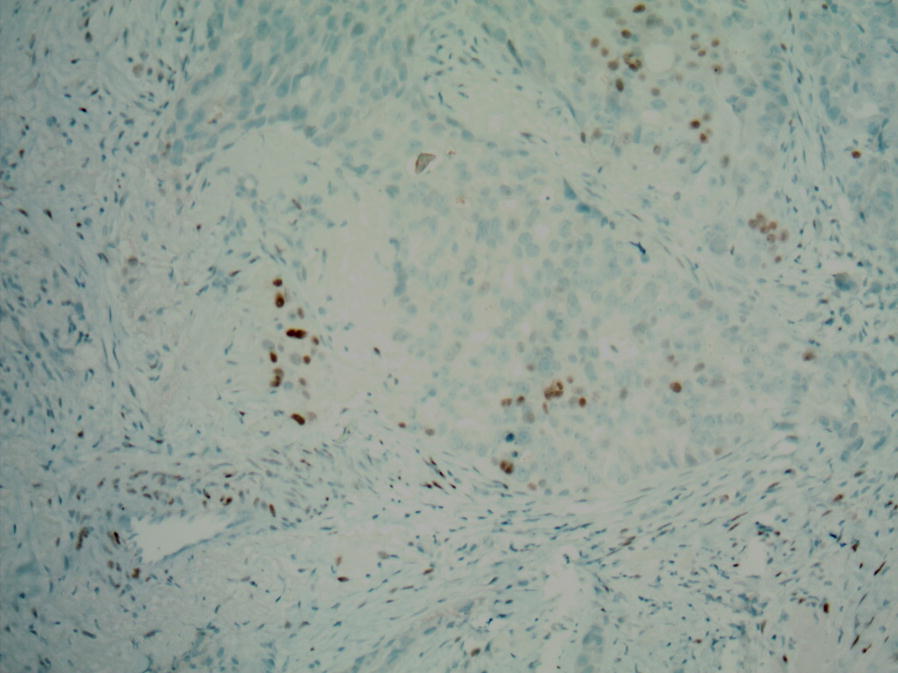
Representative area of a patient 10 (Table 4) demonstrate nuclear positivity for estrogen receptor (× 100)

In the current study, available follow-up data revealed that 4 out of 12 patients with major discrepancies were free of disease at the time of publication (Table 4). Seven patients developed recurrent or progressive disease, and for 3 of them, the recurrence was triple negative. For patients 7 and 10 of our series, the tested tissues were from metastatic sites. Patient 7 was not offered hormonal therapy. Patient 10 was offered hormonal therapy because of an ER change from negative to 15% and because her primary breast carcinoma was ER positive (Fig. 1). In only 2 of the 12 cases with major discrepancies, treatment was changed on the basis of the results of retesting and/or patient history. This finding brings into question the clinical validity of retesting triple-negative cases that are referred to our institution for treatment. It also suggests that many of these low-positive cases are still considered to be clinically hormone-receptor negative by the clinical team. In 2 studies published after the 2010 change in ER/PR positivity cutoff, Iwamoto et al. [25] and Deyarmin et al. [26] reported that these low-positive (1–9% ER/PR) tumors are molecularly closer to ER-negative tumors, suggesting that the response of these tumors to hormone-targeted therapies is suboptimal. This suggestion is in line with our clinicians’ decisions to forgo hormonal therapy in low-positive tumors.

**Table 4:** Treatment and follow-up of 12 cases with major discrepancies

Case ID	Diagnosis	Stage	Treatment	Pathologic response (if NACT)	Follow-up year (diagnosis)	Impact
1	IDC	pT1c N0	Surgery Adjuvant chemotherapy Radiation	N/A	4 (free of disease)	No hormonal therapy
2	IDC	ypT1a N0	NACT surgery Adjuvant radiation Hormone therapy	Partial	3 (free of disease)	Hormonal therapy
3	ILC	ypT3N3a	NACT surgery Adjuvant radiation	Partial	3 (developed TN Chest wall recurrence)	No hormonal therapy
4	IDC	ypT1cN1a	NACT surgery Adjuvant radiation	Almost complete	3 (free of disease)	No hormonal therapy
5	IDC	pT1cN0	Surgery Adjuvant chemotherapy	N/A	3 (free of disease)	No hormonal therapy
6	IDC	ypT1aN2a	NACT surgery Adjuvant radiation	Partial	1 (progressive metastatic disease and lost to follow-up)	No hormonal therapy
7	IDC	cT2N3M1	Palliative chemotherapy (un-resectable disease)	N/A	1 (progressive disease and lost to follow-up)	No hormonal therapy
8	IDC	pT3N0	Surgery Adjuvant chemotherapy radiation	N/A	2 (progressive TN disease)	No hormonal therapy (ER, PR, and HER2 repeated at excision were negative)
9	IDC	cT2N0	NACT (outside hospital)	N/A	N/A (lost to follow-up/ transferred care)	
10	IDC	cT2N1M1	Chemotherapy radiation (bone met)		3 (progressive ER+ disease)	Hormonal therapy (Primary breast carcinoma ER: 10%, PR: 0%)
11	IDC	ypT2N1	NACT surgery Adjuvant radiation		3 (local recurrence TN progressive disease)	No hormonal therapy
12	IDC	ypT2N1mi	NACT surgery Adjuvant radiation		2 (metastasis progressive disease: ER, PR, HER2 unknown)	No hormonal therapy

*ER* estrogen receptor, *HER2* human epidermal growth factor receptor 2, *IDC* invasive ductal carcinoma, *ILC* invasive lobular carcinoma, *N/A* not applicable, *NACT* neoadjuvant chemotherapy, *TN* triple negative

Eight (62%) of the 13 major discrepancies in the cases examined were in PR results. The clinical significance of PR has been controversial in the literature, with some studies concluding that PR does not provide significant prognostic information. They even question the relevance of the routine use of PR status in breast cancer diagnosis and treatment [27]. Nevertheless, there are sufficient published data to conclude that PR has clinically prognostic value and that the presence of PR indicates a functioning ER pathway and, therefore, an endocrine-responsive tumor [28–33]. Indeed, other studies have concluded that patients with ER-negative/PR-positive cancers and patients with ER-positive cancers respond equally to antiestrogen-based treatments, leading to the conclusion that PR status is clinically important [29, 34–36]. Antibody selection has been mentioned as a cause of false-positive PR results in some publications, especially in studies that have used the rabbit monoclonal antibody (SP2) [34]. This finding underscores the importance of implementing strict staining protocols and robust validation when this antibody is used, to avoid false-positive results.

Four of the 6 minor discrepancies in HER2 results were changes from equivocal to negative (2 + to 1 +/0). These changes do not appear to significantly affect patient treatment; however, repeat testing in equivocal cases increase costs, and these minor changes may disproportionately affect a patient’s eligibility to enroll in clinical trials [22].

This study was limited by the small number of cases examined. Therefore, we could not assess the effect of testing in reference laboratories versus community laboratories, nor could we assess the impact of the antibodies used. In addition, all tissues that were used for retesting were provided by the referring laboratories as tissue blocks or unstained slides, and we did not have control over pre-analytic variables, such as tissue fixation time. Although the same samples were used for retesting at MCC as were used in the outside labs, we do not know whether the same tissue blocks were used.

In our study, we did not evaluate the cost effectiveness of retesting. However, a previously published study on reflex testing, albeit using a different type of cases than ours, revealed significantly increased health care costs [37]. Our study findings have been of significant value to our breast pathology practice. Despite there being a 22% discrepancy rate between samples tested at our laboratory and outside laboratories, we believe that it is not cost effective to automatically retest all TNBCs, given that most of the discrepancies we found were in low ER/PR expression, which does not appear to affect treatment decisions in most cases. Therefore, based on our experience, we do not recommend reflex retesting of TNBC. At MCC we are currently retesting only those cases in which clinical and morphological tumor features are inconsistent with the diagnosis of TNBC or when the clinical team deem retesting necessary.

## Conclusions

Laboratory discordances persist in testing results for ER, PR, and HER2 in breast cancer. However, according to our findings for TNBC, major discrepancies mostly involve the reclassification of tumors as ER/PR low positive. Previously published studies have concluded that the majority of low-positive ER/PR tumors are closer in behavior to hormone receptor–negative carcinomas [26]. Therefore; these discrepancies may not be of major clinical significance at least in our setting where the changes did not appear to significantly affect treatment decisions. Regardless, accurate testing for ER, PR and HER2 continues to be the one of the most important steps in breast cancer diagnosis, treatment and prognosis.

## Data Availability

The datasets for the current study is available from the corresponding author on reasonable request.

## References

[1] Diaz LK, Sneige N (2005). Estrogen receptor analysis for breast cancer: current issues and keys to increasing testing accuracy. Adv Anat Pathol.

[2] Gown AM (2008). Current issues in ER and HER2 testing by IHC in breast cancer. Mod Pathol.

[3] Liedtke C, Mazouni C, Hess KR (2008). Response to neoadjuvant therapy and long-term survival in patients with triple-negative breast cancer. J Clin Oncol.

[4] Schmadeka R, Harmon BE, Singh M (2014). Triple-negative breast carcinoma: current and emerging concepts. Am J Clin Pathol.

[5] Boichuk S, Galembikova A, Sitenkov A (2017). Establishment and characterization of a triple negative basallike breast cancer cell line with multidrug resistance. Oncol Lett.

[6] Shao F, Sun H, Deng C-X (2017). Potential therapeutic targets of triple-negative breast cancer based on its intrinsic subtype. Oncotarget.

[7] Carey LA, Dees EC, Sawyer L (2007). The triple negative paradox: primary tumor chemosensitivity of breast cancer subtypes. Clin Cancer Res.

[8] Precht LM, Lowe KA, Atwood M, Beatty JD (2010). Neoadjuvant chemotherapy of breast cancer: tumor markers as predictors of pathologic response, recurrence, and survival. Breast J.

[9] Wolff AC, Hammond MEH, Hicks DG (2013). Recommendations for human epidermal growth factor receptor 2 testing in breast cancer: American Society of Clinical Oncology/College of American Pathologists clinical practice guideline update. Arch Pathol Lab Med.

[10] Hammond MEH, Hayes DF, Dowsett M (2010). American Society of Clinical Oncology/College of American Pathologists guideline recommendations for immunohistochemical testing of estrogen and progesterone receptors in breast cancer (unabridged version). Arch Pathol Lab Med.

[11] Wolff AC, Hammond MEH, Allison KH (2018). Human epidermal growth factor receptor 2 testing in breast cancer: American Society of Clinical Oncology/College of American Pathologists Clinical Practice Guideline Focused Update. J Clin Oncol.

[12] McCullough AE, Dell’Orto P, Reinholz MM (2014). Central pathology laboratory review of HER2 and ER in early breast cancer: an ALTTO trial [BIG 2-06/NCCTG N063D (Alliance)] ring study. Breast Cancer Res Treat.

[13] Layfield LJ, Goldstein N, Perkinson KR, Proia AD (2003). Interlaboratory variation in results from immunohistochemical assessment of estrogen receptor status. Breast J.

[14] Moatamed NA, Nanjangud G, Pucci R (2011). Effect of ischemic time, fixation time, and fixative type on HER2/neu immunohistochemical and fluorescence in situ hybridization results in breast cancer. Am J Clin Pathol.

[15] Schink JC, Trosman JR, Weldon CB (2014). Biomarker testing for breast, lung, and gastroesophageal cancers at NCI designated cancer centers. J Natl Cancer Inst.

[16] Parker RL, Huntsman DG, Lesack DW (2002). Assessment of interlaboratory variation in the immunohistochemical determination of estrogen receptor status using a breast cancer tissue microarray. Am J Clin Pathol.

[17] Perez EA, Press MF, Dueck AC (2013). Immunohistochemistry and fluorescence in situ hybridization assessment of HER2 in clinical trials of adjuvant therapy for breast cancer (NCCTG N9831, BCIRG 006, and BCIRG 005). Breast Cancer Res Treat.

[18] Paik S, Bryant J, Tan-Chiu E (2002). Real-world performance of HER2 testing—national surgical adjuvant breast and bowel project experience. J Natl Cancer Inst.

[19] Reddy JC, Reimann JD, Anderson SM, Klein PM (2006). Concordance between central and local laboratory HER2 testing from a community-based clinical study. Clin Breast Cancer.

[20] O’Malley FP, Thomson T, Julian J (2008). HER2 testing in a population-based study of patients with metastatic breast cancer treated with trastuzumab. Arch Pathol Lab Med.

[21] Roche PC, Suman VJ, Jenkins RB (2002). Concordance between local and central laboratory HER2 testing in the breast intergroup trial N9831. J Natl Cancer Inst.

[22] Rosa M, Khazai L (2018). Comparison of HER 2 testing among laboratories: our experience with review cases retested at Moffitt Cancer Center in a two-year period. Breast J.

[23] Pinder SE, Campbell AF, Bartlett JM (2017). Discrepancies in central review re-testing of patients with ER-positive and HER2-negative breast cancer in the OPTIMA prelim randomised clinical trial. Br J Cancer.

[24] Badve SS, Baehner FL, Gray RP (2008). Estrogen- and progesterone-receptor status in ECOG 2197: comparison of immunohistochemistry by local and central laboratories and quantitative reverse transcription polymerase chain reaction by central laboratory. J Clin Oncol.

[25] Iwamoto T, Booser D, Valero V (2012). Estrogen receptor (ER) mRNA and ER-related gene expression in breast cancers that are 1% to 10% ER-positive by immunohistochemistry. J Clin Oncol.

[26] Deyarmin B, Kane JL, Valente AL (2013). Effect of ASCO/CAP guidelines for determining ER status on molecular subtype. Ann Surg Oncol.

[27] Hefti MM, Hu R, Knoblauch NW (2013). Estrogen receptor negative/progesterone receptor positive breast cancer is not a reproducible subtype. Breast Cancer Res.

[28] Bardou V-J, Arpino G, Elledge RM, Osborne CK, Clark GM (2003). Progesterone receptor status significantly improves outcome prediction over estrogen receptor status alone for adjuvant endocrine therapy in two large breast cancer databases. J Clin Oncol.

[29] Colomer R, Beltran M, Dorcas J (2005). It is not time to stop progesterone receptor testing in breast cancer. J Clin Oncol.

[30] Fuqua SA, Cui Y, Lee AV, Osborne CK, Horwitz KB (2005). Insights into the role of progesterone receptors in breast cancer. J Clin Oncol.

[31] Kornaga EN, Klimowicz AC, Guggisberg N (2016). Evaluation of three commercial progesterone receptor assays in a single tamoxifen-treated breast cancer cohort. Mod Pathol.

[32] Knutson TP, Lange CA (2014). Tracking progesterone receptor-mediated actions in breast cancer. Pharmacol Ther.

[33] Osborne CK, Schiff R, Arpino G, Lee AS, Hilsenbeck V (2005). Endocrine responsiveness: understanding how progesterone receptor can be used to select endocrine therapy. Breast J.

[34] Ibrahim M, Dodson A, Barnett S, Fish D, Jasani B, Miller K (2008). Potential for false-positive staining with a rabbit monoclonal antibody to progesterone receptor (SP2): findings of the UK National External Quality Assessment Scheme for Immunocytochemistry and FISH highlight the need for correct validation of antibodies on introduction to the laboratory. Am J Clin Pathol.

[35] Ciocca DR, Elledge R (2000). Molecular markers for predicting response to tamoxifen in breast cancer patients. Endocrine.

[36] Fan Y, Ding X, Xu B (2015). Prognostic significance of single progesterone receptor positivity: a comparison study of estrogen receptor negative/progesterone receptor positive/Her2 negative primary breast cancer with triple negative breast cancer. Medicine (Baltimore).

[37] VandenBussche CJ, Cimino-Mathews A, Park BH, Emens LA, Tsangaris TN, Argani P (2015). Reflex estrogen receptor/progesterone receptor/human epidermal growth factor receptor 2 (ER/PR/Her2) analysis of breast cancers in needle core biopsy specimens dramatically increases health care costs. Am J Surg Pathol.

